# Supplementary material for: Minimal Determinants for Lifelong Antiviral Antibody Responses in Mice from a Single Exposure to Virus-like Immunogens at Low Doses
Source: Vaccines (Basel). 2024 Apr 11;12(4):405. doi: 10.3390/vaccines12040405 (PMC11054763; doi:10.3390/vaccines12040405)
Supplement: Supplementary file 1 [file vaccines-12-00405-s001.zip › vaccines-2914626-supplementary.pdf]

The supporting information include:

Supporting Figs. S1 and S2

Fig. S1. Indicator of affinity maturation that is largely independent of sera IgG concentrations.

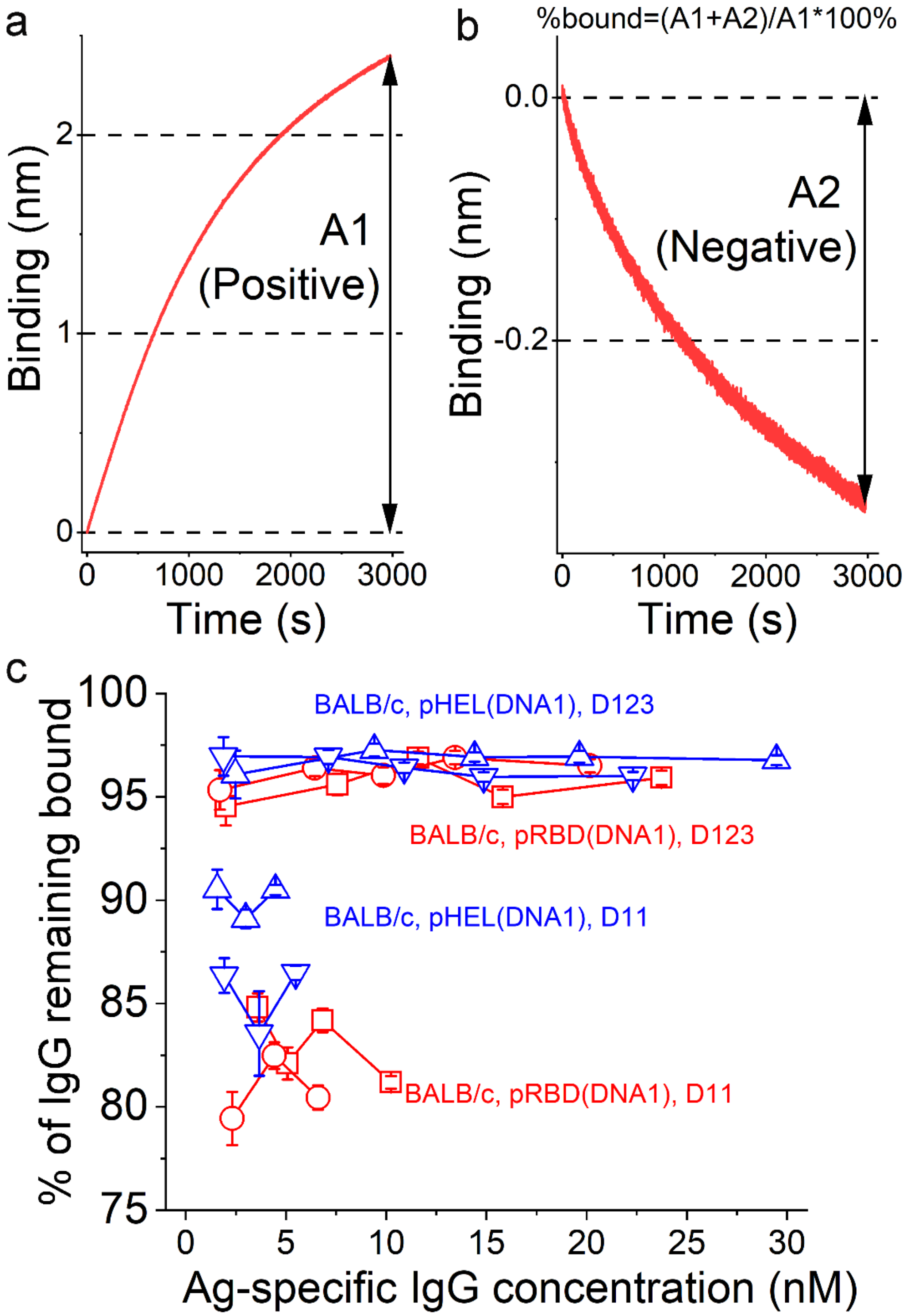

(**a**) A representative serum IgG binding curve showing the definition of binding amplitude, A1, that is measured from BioLayer interferometry experiments. A1 is the difference in the binding signal between the end and the beginning of a binding curve, which takes a positive value with a

unit of nm. (**b**) The corresponding dissociation curve from (a) showing the definition of dissociation amplitude, A2, that is measured from BioLayer interferometry experiments. A2 is the difference in the binding signal between the end and the beginning of a dissociation curve, which takes a negative value with a unit of nm. The percentage of IgG remaining bound at the end of dissociation is calculated as indicated on top of panel (b). (**c**) The percentage of IgG that remains bound at the end of dissociation was plotted as a function of the Ag-specific IgG concentration. For each color and symbol, the different concentrations were realized by dilution of the sera and then BioLayer interferometry experiments were conducted. This was done for several serum samples from BALB/c mice immunized with the agents as noted in the figure. Specifically, red squares, red circles, upper triangles, and down triangles are for pRBD(DNA1)3, pRBD(DNA1)4, pHEL(DNA1)1, and pHEL(DNA1)2, respectively. The dose of RBD was 0.24 µg per animal and the dose of HEL was 0.1 µg per animal. The concentrations for Ag-specific IgG were determined using magnetic beads capture as described in Materials and Methods. N=4 for each Ag-specific IgG concentration in (c).

Fig. S2. A representative standard curve from quantitative western blots to determine the concentration of Ag-specific IgG in mouse sera.

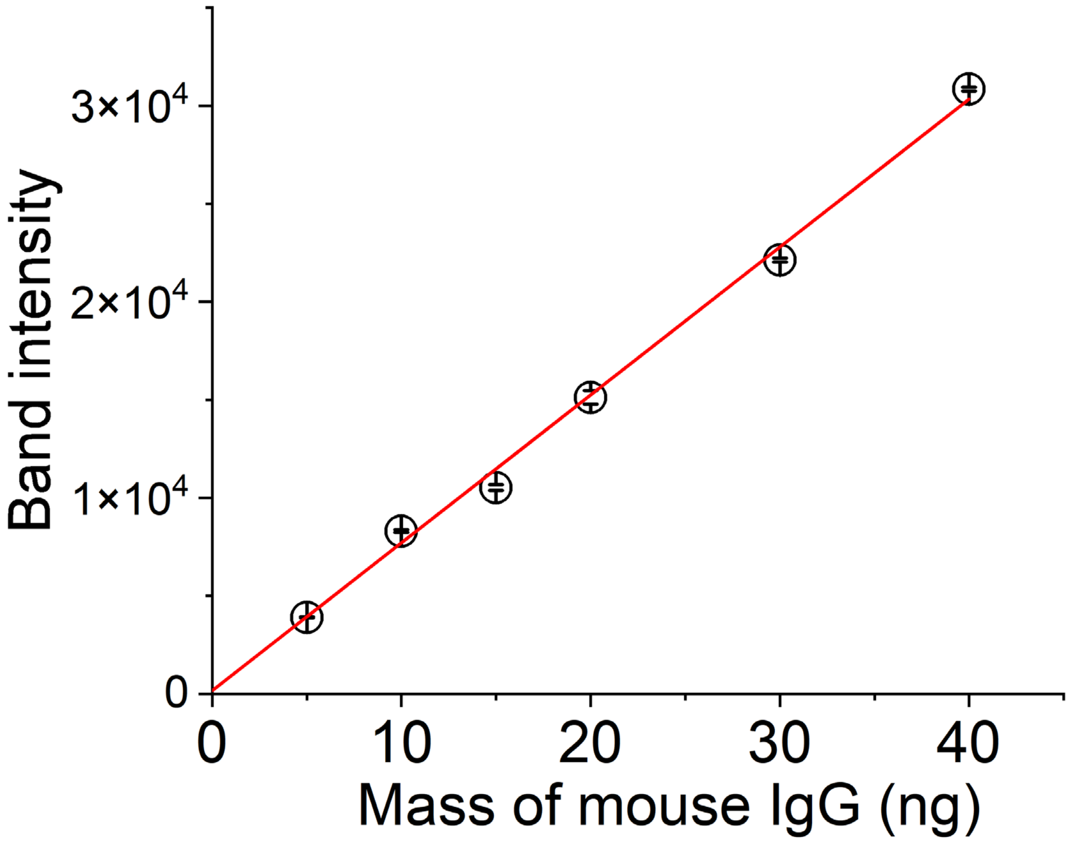

We used mAb1, a commercial monoclonal mouse IgG (BioLegend CAT#944803) as the known reference to construct a standard curve on the same western blot on which Ag-specific IgG antibodies captured by streptavidin magnetic beads were loaded. The band intensity was quantified using ImageJ and plotted against the loaded references (N=3 for each data point). A linear regression (the straight red line) was then used to quantitate the mass of Ag-specific IgG in serum samples. The adjusted R-square value from the linear regression as shown above is 0.998. The mass was then used to calculate the concentration of the IgG based on volume dilutions.
